# Traumatic Pseudoaneurysm of the Ascending Cervical Artery Treated with N-butyl Cyanoacrylate Embolization: A Case Report and Review of the Literature

**DOI:** 10.7759/cureus.6276

**Published:** 2019-12-02

**Authors:** Torin W Karsonovich, John C Hawkins, Ajeet Gordhan

**Affiliations:** 1 Neurological Surgery, Advocate BroMenn Medical Center, Normal, USA; 2 Neurointerventional Radiology and Surgery, OSF HealthCare, Bloomington, USA

**Keywords:** pseudoaneurysm, thyrocervical trunk, ascending cervical artery, n-butyl cyanoacrylate

## Abstract

Pseudoaneurysms of the thyrocervical trunk and its branches are commonly iatrogenic in nature; however, trauma is often an inciting mechanism. Open surgical repair was considered the main treatment modality until recent advances in endovascular therapy proved to be a viable treatment option. We report a case of a traumatic pseudoaneurysm arising from the ascending cervical artery with an associated arteriovenous fistula (AVF) that was treated using n-butyl cyanoacrylate (NBCA) embolization. The use of a liquid embolysate such as NBCA provided an efficient and effective means of achieving both pseudoaneurysm occlusion and AVF disconnection.

## Introduction

Pseudoaneurysms are uncommon complications of traumatic or iatrogenic arterial injuries that, by definition, are transmural and contained by a thin layer of surrounding connective tissue. Oftentimes, these lesions can be accompanied by arteriovenous fistulae (AVFs). When pseudoaneurysms involve the thyrocervical trunk and its branches, the usual cause is iatrogenic injury while attempting an internal jugular catheterization [1]. Historically, pseudoaneurysms and AVFs have been treated with open surgery, but endovascular intervention has gained attention in recent years. We present a case of a traumatic pseudoaneurysm and AVF of the ascending cervical artery from the thyrocervical trunk that was treated with n-butyl cyanoacrylate (NBCA). To our knowledge, this form of treatment has never been reported for pseudoaneurysms arising from the branch vascularity of the thyrocervical trunk.

## Case presentation

**Figure 1:**
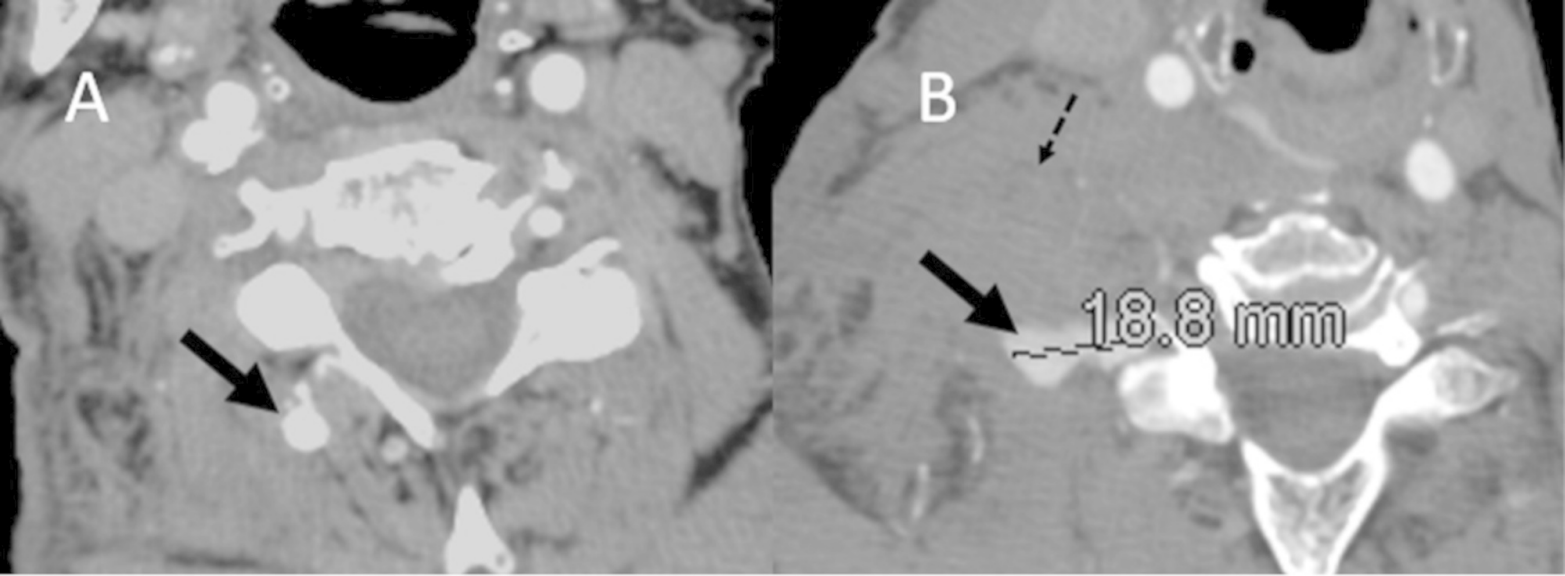
(A) Axial section CT angiography demonstrating a focal prominence at the distal aspect of the ascending cervical artery (black arrow). (B) Axial section CT angiography demonstrating extravascular contrast deep within the right lateral neck in conjunction with a probable right lateral neck hematoma (dotted arrow).

**Figure 2:**
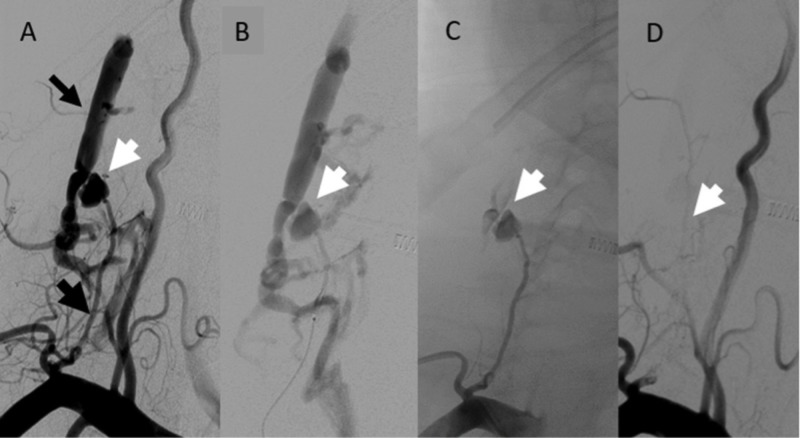
(A) Digital subtraction angiography of the right subclavian artery demonstrating a diminutive ascending cervical artery arising from the thyrocervical trunk (thick black arrow). A pseudoaneurysm at the distal aspect of the ascending cervical artery (white arrow) demonstrates a fistulous connection to a cervical vein (thin black arrow). (B) Micro-catheter super-selective angiography of the ascending cervical artery demonstrating no dangerous collateral to the vertebral or ascending pharyngeal arteries. (C) NBCA cast obliterating the pseudoaneurysm with cast penetration into the fistulous connection. (D) Right subclavian artery injection post-embolization with complete disconnection of the AVF and with patency of the right vertebral artery. NBCA, n-butyl cyanoacrylate; AVF, arteriovenous fistula.

The patient is an 80-year-old female with a history of hypertension, chronic obstructive pulmonary disease, congestive heart failure, and diabetes who presented to the emergency department after suffering a ground-level fall at home. She was neurologically intact, with complaints of localized pain in the right clavicular region. She suffered a right-sided comminuted fracture of the distal clavicle with displacement. A computed tomography angiogram (CTA) demonstrated an aneurysmal prominence of the right ascending cervical artery with active contrast extravasation, measuring 18 mm, and a hematoma at the level of C6 (Figure 1). She was initially observed with external pressure in the right anterior neck region with a plan for a repeat CTA in 12-24 hours. Over the next four hours, she had a progressive decline in her hemoglobin level from 13.9 g/dL to 10.3 g/dL without associated hemodynamic instability. Angiographic intervention was recommended at this time. This revealed a pseudoaneurysm at the distal aspect of the right ascending cervical artery arising from the thyrocervical artery in conjunction with an unexpected low-flow AVF. With guided catheter placement at the origin of the thyrocervical trunk, a micro-catheter was navigated into the ascending cervical artery just proximal to the pseudoaneurysm. Super-selective micro-catheter angiography identified no anomalous arterial supply to the ipsilateral vertebral artery or the ascending pharyngeal artery. Embolic occlusion of the pseudoaneurysm as well as disconnection of the AVF was achieved using NBCA (Figure 2). She tolerated the procedure well and was discharged home on hospital day 3, with stabilization of her hemoglobin level.

## Discussion

The thyrocervical trunk is the second superiorly directed branch to exit the subclavian artery. The thyrocervical trunk and its branches, including the ascending cervical artery, are well protected by the surrounding soft tissue and, therefore, are relatively protected from external injury. Most thyrocervical artery pseudoaneurysms and AVFs occur as a result of penetrating traumas and iatrogenic needle punctures, but these injuries in this location are rare [2]. Our patient’s injury occurred as a result of a blunt trauma following a fall, which is an exceedingly rare presentation. Her symptoms of localized pain and supraclavicular mass are expected, as pseudoaneurysms typically present as a pulsatile mass with a possible associated bruit [2].

Pseudoaneurysms may spontaneously disappear due to thrombosis, but they can also rupture, causing massive hemorrhage, or increase in size, leading to a mass effect on the surrounding vascular and neurologic structures [3]. It is because of these reasons that emergent catheter-based angiography and subsequent embolic occlusion was performed in our patient. Evaluation of a suspected pseudoaneurysm or AVFs may be performed with CTA, which carries a sensitivity and specificity of more than 95% [4]. Duplex sonography has also been shown to provide similar reliability to CTA and MRA (magnetic resonance angiography), but spatial resolution and details of the associated vasculature are limited [5]. Digital subtraction angiography is considered the gold standard and provides vital information regarding collateral vascularization and thyrocervical trunk anastomoses to important neurologic structures. The ascending cervical artery, in particular, has been associated with anastomoses to the vertebral, occipital, and ascending pharyngeal arteries, providing perfusion to the vertebral bodies, spinal cord, and meninges [4]. A feared complication of embolic therapy to the thyrocervical trunk or its branches is an anterior spinal cord infarct, potentially causing complete motor paralysis and the loss of pain and temperature sensation below the lesion, along with possible bowel and sexual dysfunction [4]. Involvement of the basilar artery through the anastomoses between the vertebral artery and the ascending cervical artery can lead to coma or death. For this reason, super-selective micro-catheter angiography is necessary prior to endovascular embolic occlusion.

Treatment of thyrocervical trunk pseudoaneurysms and AVFs has classically been approached through open surgical repair, but this is associated with significant morbidity and mortality, longer recovery, and higher costs [5]. Ultrasound-guided compression and thrombin injection are new alternatives for the treatment of superficial pseudoaneurysms. Ultrasound-guided percutaneous thrombin injection has shown to be successful in more than 90% of cases reported [6]. However, with deeper and more complex lesions, such as those with AVFs, a standardized treatment has not been elucidated.

Endovascular treatments have emerged, providing reduced recovery time, lower cost, and decrease in operative morbidity [5]. This modality is applied with the goal of separating the pseudoaneurysm from its circulation and has been successfully described in the thyrocervical trunk using detachable coils [2, 5]. Pseudoaneurysms in other areas of the vasculature have been treated with other endovascular methods such as with flow-diverting stents [7], Onyx [8], and NBCA [9]. NBCA was considered in our case as it is a permanent adhesive liquid embolic agent with a low viscosity that allows for deeper small vessel penetration and can be performed in a much quicker and less expensive manner than detaching several coils [9].

## Conclusions

To our knowledge, NBCA embolization to treat a blunt trauma related fistulous pseudoaneurysm of the ascending cervical artery arising from the thyrocervical trunk has not been reported. Due to the presence of several important anastomotic links from the thyrocervical trunk, the prevention of aberrant embolization is crucial in avoiding significant morbidity or mortality. Catheter-based angiography with super-selective micro-catheter angiography is, therefore, imperative prior to endovascular occlusion of such lesions. The use of a liquid embolysate such as NBCA provided an efficient and effective means of achieving both pseudoaneurysm occlusion and AVF disconnection.
